# A comprehensive model for familial breast cancer incorporating BRCA1, BRCA2 and other genes

**DOI:** 10.1038/sj.bjc.6600008

**Published:** 2002-01-07

**Authors:** A C Antoniou, P D P Pharoah, G McMullan, N E Day, M R Stratton, J Peto, B J Ponder, D F Easton

**Affiliations:** CRC Genetic Epidemiology Unit, Institute of Public Health, Strangeways Research Laboratory, Worts Causeway, University of Cambridge, Cambridge CB1 8RN, UK; CRC Human Cancer Genetics Research Group, University of Cambridge, Cambridge CB1 8RN, UK; High Performance Computing Facility, Cavendish Laboratory, University of Cambridge, Cambridge CB1 8RN, UK; Department of Public Health and Primary Care, University of Cambridge, Cambridge CB1 8RN, UK; Institute of Cancer Research, Sutton, Surrey, UK

**Keywords:** segregation analysis, BRCA3, Polygenes, high-risk families, population studies

## Abstract

In computing the probability that a woman is a BRCA1 or BRCA2 carrier for genetic counselling purposes, it is important to allow for the fact that other breast cancer susceptibility genes may exist. We used data from both a population based series of breast cancer cases and high risk families in the UK, with information on BRCA1 and BRCA2 mutation status, to investigate the genetic models that can best explain familial breast cancer outside BRCA1 and BRCA2 families. We also evaluated the evidence for risk modifiers in BRCA1 and BRCA2 carriers. We estimated the simultaneous effects of BRCA1, BRCA2, a third hypothetical gene ‘BRCA3’, and a polygenic effect using segregation analysis. The hypergeometric polygenic model was used to approximate polygenic inheritance and the effect of risk modifiers. BRCA1 and BRCA2 could not explain all the observed familial clustering. The best fitting model for the residual familial breast cancer was the polygenic, although a model with a single recessive allele produced a similar fit. There was also significant evidence for a modifying effect of other genes on the risks of breast cancer in BRCA1 and BRCA2 mutation carriers. Under this model, the frequency of BRCA1 was estimated to be 0.051% (95% CI: 0.021–0.125%) and of BRCA2 0.068% (95% CI: 0.033–0.141%). The breast cancer risk by age 70 years, based on the average incidence over all modifiers was estimated to be 35.3% for BRCA1 and 50.3% for BRCA2. The corresponding ovarian cancer risks were 25.9% for BRCA1 and 9.1% for BRCA2. The findings suggest that several common, low penetrance genes with multiplicative effects on risk may account for the residual non-BRCA1/2 familial aggregation of breast cancer. The modifying effect may explain the previously reported differences between population based estimates for BRCA1/2 penetrance and estimates based on high-risk families.

*British Journal of Cancer* (2002) **86**, 76–83. DOI: 10.1038/sj/bjc/6600008 www.bjcancer.com

© 2002 The Cancer Research Campaign

## 

Of the breast cancer susceptibility genes which have been identified to date, BRCA1 and BRCA2 are the most important ‘high risk’ genes accounting for the majority of families with multiple cases of breast and ovarian cancer. Other breast cancer susceptibility alleles include mutations in the TP53 gene (Malkin *et al*, 1990), mutations in the PTEN gene (Nelen *et al*, 1996) and rare HRAS1 mutations (Krontiris *et al*, 1993). There is also evidence that heterozygous carriers of the ATM gene are at increased risks of breast cancer (Easton, 1994; Swift *et al*, 1991; Olsen *et al*, 2001). But even if the effects of all the identified genes are taken together, it is estimated that they can explain only 20–25% of the familial relative risk for breast cancer (Easton, 1999; Rebbeck, 1999). Evidently other breast cancer susceptibility genes may exist. However, it is unclear whether the residual familial aggregation is due to rare mutations conferring a high risk of the disease, similar to BRCA1/2, or to common but lower risk variants.

In genetic counselling it is important to have an accurate genetic model on which to base estimates of mutation carrier probabilities and cancer risks. Such a model needs to incorporate both the effects of both the known susceptibility genes (BRCA1 and BRCA2) and of other possible breast cancer susceptibility genes. The existing genetic models are either based on a single susceptibility gene (Claus *et al*, 1991) or take into account only the effects of BRCA1 and BRCA2 (Parmigiani *et al*, 1998) and hence they do not describe the familial breast cancer adequately.

In this study we have combined data from a population-based series of breast cancer cases with data from high risk families, to investigate the genetic models that can explain familial breast cancer outside BRCA1 and BRCA2 families, and evaluate the evidence for risk modifiers in BRCA1 and BRCA2 mutation carriers. In a previous analysis of the population based Anglian Breast Cancer study (Antoniou *et al*, 2001), we have demonstrated that the familial aggregation of breast cancer can be adequately explained by the effects of BRCA1, BRCA2 and a polygenic component (i.e. a large number of low risk polymorphisms acting multiplicatively). Here we investigate how these models fit data on high risk families, and evaluate the modifying effects of other genes in BRCA1/2 carriers.

## MATERIALS AND METHODS

### Anglian Breast Cancer (ABC) study

The data collection for this study is described in more detail elsewhere (The Anglian Breast Cancer Study Group (2000); Antoniou *et al*, 2001). One thousand four hundred and eighty four cases diagnosed with breast cancer under the age 55 years were identified in the region served by the Anglian Cancer Registry between 1991 and 1996. The cases were invited to provide blood sample and to complete an epidemiological questionnaire including information on family history of cancer in all first degree relatives. Genomic DNA, extracted from the blood, was screened for BRCA1 and BRCA2 mutations by Conformation Sensitive Gel Electrophoresis (CSGE), and mutations were confirmed by sequencing.

### Multiple case families

One hundred and fifty six families were ascertained in response to national publicity in the UK and by referral by oncologists or general practitioners. Each family contained two or more breast cancer cases, at least one of which was diagnosed under age 50 years. Cancer occurrence and follow up was recorded on all family members. At least one individual from each family had DNA samples analyzed for BRCA1 and BRCA2 mutations by CSGE. These families are referred to as the ‘B’ families.

### Statistical methods

The analysis was based on breast and ovarian cancer occurrence in the combined dataset of the ‘B’ families and the ABC study. All individuals were censored at age 80 years, the age of their first cancer or their age of death, whichever occurred first. Thus, only information on the first cancer was included in the analysis. Individuals known to have died but with unknown age at death were censored at age last at follow up or at age 70 years, whichever was smaller. If it was known that an individual had developed breast or ovarian cancer, but the age at diagnosis was unknown, we treated the age at interview or age at death, whichever was smaller, as the age of cancer. Individuals with no age information were treated as lost at follow up and were censored at age zero years. Females who had had prophylactic oophorectomy were censored for both breast and ovarian cancer at the age of the surgery since oophorectomy is known to reduce the risk of breast cancer as well as ovarian cancer. Females who had had mastectomy were censored for breast cancer at the age of the surgery. The analyses were implemented using the computer program MENDEL (Lange and Weeks, 1988) which we parallelized to run on a Hitachi SR2201 parallel computer of the University of Cambridge High Performance Computing Facility.

### Models

We modelled the simultaneous effects of BRCA1, BRCA2, a third hypothetical gene BRCA3 which was assumed to have increased risks of breast cancer, and a polygenic effect. The models for this analysis are described in detail elsewhere (Antoniou *et al*, 2001). Briefly, the breast cancer incidence rate λ(*t*) was assumed to be based on the Cox model (Cox and Oakes, 1984), λ(*t*)=λ_0_(*t*)exp(*G*+*P*). λ_0_(*t*) is the baseline incidence rate, *G* depends on the major genotype and *P* is the polygenic component, which is assumed to be normally distributed with zero mean and variance 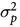. The polygenic component *P* was assumed to act in BRCA1 and BRCA2 carriers as well as non-carriers. However, we also fitted models in which *P* had no effect in BRCA1/2 carriers, and models in which the variance of the polygenic ‘modifying’ component was allowed to take a different value 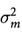. The Hypergeometric Polygenic Model (HPM) (Lange, 1997a,b) was used to approximate the polygenic and modifying components, where *P* was approximated by


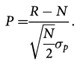


*R* has a binomial distribution (2*N*, ½) according to the HPM and *N* is the number of loci used in the HPM, which was set equal to 3 in our analyses.

The ovarian cancer incidence rates were based on a similar model, but we assumed that the polygenotype and the modifying genes did not have any effect on the ovarian cancer risks. BRCA3 carriers were assumed to develop ovarian cancer according to the general population incidence rates. Breast and ovarian cancers were assumed to occur independently conditional on genotype.

The overall breast and ovarian cancer incidence rates were constrained to agree with the national incidence rates for England and Wales (1983–1987). The implementation of these constraints is described elsewhere (Antoniou *et al*, 2001). In order to estimate the BRCA1 and BRCA2 cancer risks, we assumed that the observed incidence rates for BRCA1 and BRCA2 mutation carriers were a constant multiple (*RR*) of the Breast Cancer Linkage Consortium (BCLC) rates (Easton *et al*, 1993; Ford *et al*, 1998). We then investigated the effect of allowing the relative risks (*RR*) to vary with age. For the models allowing for the modifying effect of other genes on the breast cancer risks of BRCA1 and BRCA2, we assumed that the observed incidence rate was the average over all possible modifying effects.

In the ABC families, only the index case was tested for BRCA1 and BRCA2 mutations. In the ‘B’ families, mutation testing was first performed on an index affected individual. If a mutation was found, other relatives were then screened for the same mutation. For the index cases we assumed that the sensitivity of the mutation testing was 64%, previously estimated by the Breast Cancer Linkage Consortium for similar methodologies (Ford *et al*, 1998). For any other relatives the mutation screening was assumed to be 100% sensitive and 100% specific. In the ABC study only the index patient was tested, and the screening sensitivity was assumed to be 64%.

### Estimation

The genetic models were specified in terms of the breast and ovarian cancer incidence rates in BRCA1/2 carriers, the allele frequencies, the number of loci (*N*) in the HPM and the standard deviation for the polygenic and modifying components, σ*_p_* and σ_*m*_. Maximum likelihood estimation was used to estimate the parameters. For the ABC families we maximized the conditional likelihood of observing the disease phenotypes and mutation status given the disease phenotype of the index case. For the ‘B’ families, which were ascertained on the basis of multiple affected individuals, we conditioned on the disease phenotypes of all family members.

The variances of the parameters were estimated by inverting the observed information matrix. To compute confidence intervals we used transformed values of the parameters which provide estimates which are normally distributed. Gene frequencies were transformed using the logit function log 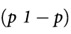, while for relative risks and 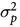 and 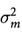 we used the log transformation.

### Goodness of fit

The goodness of fit of the models was tested using likelihood based tests. Starting from a ‘sporadic’ model (i.e. only the BRCA1 and BRCA2 effects taken into account), we tested if the addition of parameters improved the fit significantly. The models were constructed by adding parameters that were significant in a stepwise fashion. Non-nested models were compared using the Akaike Information Criterion (Akaike, 1974):


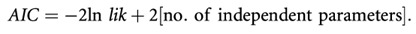


The model that minimizes AIC was chosen (Elston, 1990). For the best fitting models we also compared the predicted number of mutations in index cases with the number observed.

### Carrier probabilities

To calculate the number of mutations predicted by each model we first computed the probability that the index case was a mutation carrier, on the basis of family history (FH) of breast or ovarian cancer. Thus, the probability π_*j*__1_ that the index case in family *j* was a BRCA1 carrier was given by:


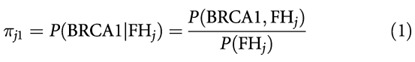



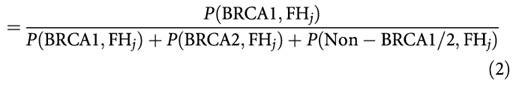


These probabilities were computed in MENDEL as the ratio of the likelihood of observing the family with the BRCA1 mutation divided by the likelihood of observing all possibilities. The total number of BRCA1 mutations was then obtained by summing π_*j*__1_ over all families. BRCA2 predictions were computed similarly.

## RESULTS

### ABC families

Table 1

**Table 1:**
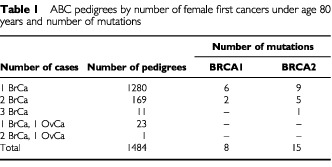
ABC pedigrees by number of female first cancers under age 80 years and number of mutations

summarizes the pedigrees by the number of breast and ovarian cancer cases diagnosed under age 80 years. One thousand two hundred and eighty probands did not have any family history of breast or ovarian cancer in the first degree relatives. One hundred and sixty-nine cases had one first degree relative diagnosed with breast cancer and 11 had two first degree relatives diagnosed with breast cancer. Twenty-three cases had one relative that developed ovarian cancer under age 80 years, and one case had one breast cancer case and one ovarian cancer case in first degree relatives. There were some differences between the present analysis and the previous analysis of the ABC dataset (Antoniou *et al*, 2001), with regard to the number of mutations. Three mutations previously thought to be disease causing have been reclassified as non-disease causing, while five new mutations have been found by reevaluating the CSGE gels. In total there were eight BRCA1 mutations and 15 BRCA2 mutations. Six of the BRCA1 mutation carriers did not have any family history of breast or ovarian cancer in first degree relatives and two had a relative diagnosed with breast cancer under age 80 years. Nine BRCA2 mutation carriers had no family history of breast or ovarian cancer, five had one relative that developed breast cancer and one had two first degree relatives diagnosed with breast cancer. Mutations were not identified in any of the 24 index cases with a family history of ovarian cancer.

### ‘B’ families

Table 2

**Table 2:**
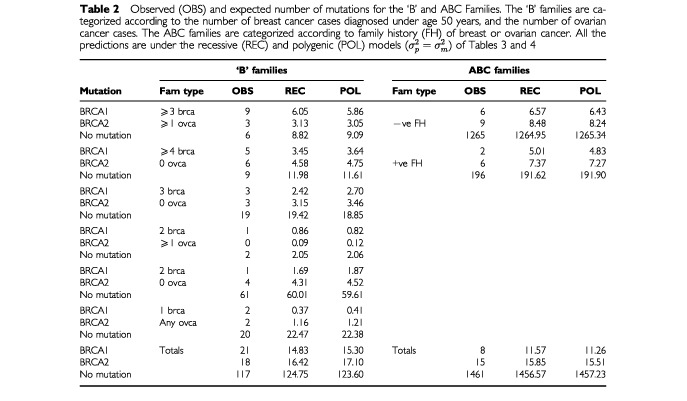
Observed (OBS) and expected number of mutations for the ‘B’ and ABC Families. The ‘B’ families are categorized according to the number of breast cancer cases diagnosed under age 50 years, and the number of ovarian cancer cases. The ABC families are categorized according to family history (FH) of breast or ovarian cancer. All the predictions are under the recessive (REC) and polygenic (POL) models (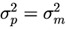) of Tables 3 and 4

shows the families grouped by the number of breast cancer cases diagnosed under age 50 years and the number of ovarian cancer cases diagnosed under age 80 years. Eighteen families had at least three breast cancer cases diagnosed under age 50 years and at least one ovarian cancer case. Twenty families had at least four breast cancer cases diagnosed under age 50 years and no ovarian cancer cases. Twenty five families had only three breast cancer cases diagnosed under age 50 years, three families had two breast cancer cases and at least one ovarian cancer case, 66 families had two breast cancer cases under age 50 years and 24 families had one breast cancer case diagnosed under age 50 years. In total there were 618 cases of breast cancer diagnosed at any age and 32 cases of ovarian cancer. 21 (13%) of the index cases were tested positive for BRCA1 mutations and 18 (11.5%) tested positive for BRCA2 mutations. The majority of the mutations were in families with at least three breast cancer cases. In total, 318 individuals were tested for BRCA1 and BRCA2 mutations (including the index cases). Fifty seven BRCA1 mutation carriers and 74 BRCA2 mutation carriers were identified.

### Segregation analysis

#### Major gene models

Table 3

**Table 3:**
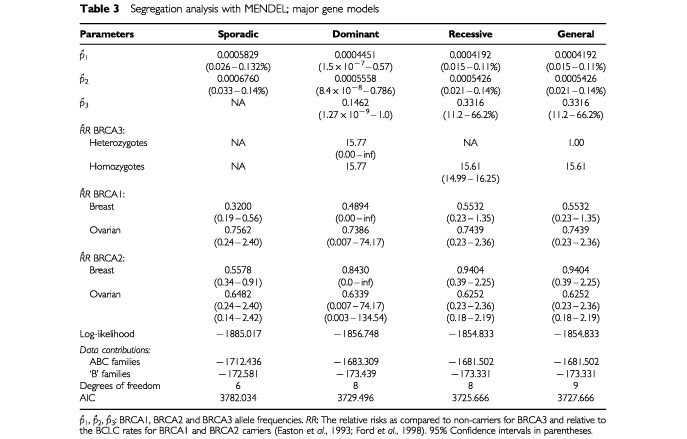
Segregation analysis with MENDEL; major gene models

summarizes the results for the major gene models for BRCA3 incorporating BRCA1 and BRCA2 mutations into the models but no allowance for polygenic effects. In these, we assumed that BRCA3 mutations conferred a constant relative risk over all age groups relative to the background incidence rates. The major gene models included a dominant, a recessive and a general model where the homozygous and heterozygous carriers were assumed to have different relative risks. All three models fitted significantly better than the sporadic model (*P*<0.0001). The general model converged to the recessive model which had the lowest AIC among these models. Under the recessive model, BRCA3 was estimated to have an allele frequency of 33% with a cumulative breast cancer risk of 27.6% by age 70 years in the homozygous carriers. The mutation frequencies were estimated to be 0.042% for BRCA1 and 0.054% for BRCA2. The breast cancer risk by age 70 years was estimated to be 49.6% for BRCA1 and 82.2% for BRCA2. The corresponding ovarian cancer risks were 32.8% for BRCA1 and 17.8% for BRCA2.

#### Polygenic models

The results for the polygenic models are shown in Table 4

**Table 4:**
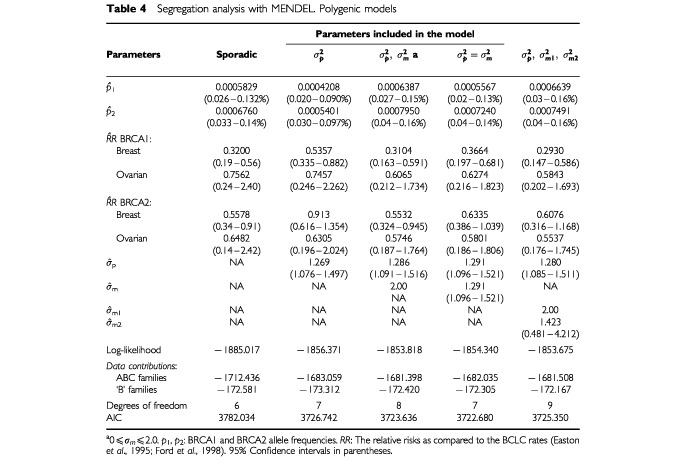
Segregation analysis with MENDEL. Polygenic models

. The inclusion of the polygenic component (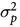) improved the fit significantly over the sporadic model (*P*<0.0001). There was also significant evidence for a modifying effect on the breast cancer risks of BRCA1 and BRCA2 mutation carriers (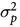, 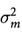, *P*=0.0238). But there was no evidence for a difference between the ‘modifying variance’ 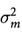 and the polygenic variance in non carriers. Thus the model with a single polygenic variance term was the most parsimonious. We also tested a model with different modifying variances in BRCA1 and BRCA2 carriers. Under this model, the BRCA1 modifying variance reached an artificial upper boundary of 2.0, but the BRCA2 modifying variance converged to a lower value (1.423). This was due to the absence of family history of breast cancer in BRCA1 mutation carriers in the ABC families. However, the model was not a significantly better fit over the model with a common variance in BRCA1 and BRCA2 carriers.

#### BRCA1 and BRCA2 risks

Under the most parsimonious model, (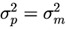) the allele frequency for BRCA1 mutations in the general population was estimated to be 0.056% (95% CI: 0.023–0.132%) and the allele frequency for BRCA2 mutations was 0.072% (95% CI: 0.037–0.143%). The standard deviation for the polygenic and modifying effects was estimated to be 1.291 (95% CI: 1.096–1.521). The relative risks of breast and ovarian cancer in BRCA1 and BRCA2 carriers, relative to the BCLC risks (Easton *et al*, 1993; Ford *et al*, 1998) were all estimated to be less than one. The cumulative breast cancer risk for a BRCA1 mutation carrier, based on the observed incidence, was estimated to be 36.5% by age 70 years and the ovarian cancer risk 28.5%. The cumulative breast cancer risk for a BRCA2 mutation carrier was estimated to be 68.7% and the corresponding ovarian cancer risk was 16.6%.

To estimate more precisely the shape of the incidence curves for BRCA1 and BRCA2 mutation carriers, we fitted a model where separate breast cancer relative risks were assumed for ages 20–39, 40–49, 50–59 and 60–79 years, for both BRCA1 and BRCA2 mutation carriers. We also allowed for separate ovarian cancer relative risks in the age groups 30–49, 50–59, and 60–69 years for BRCA1 and in the age groups 30–49 and 50–69 years for BRCA2. The number of ovarian cancer cases were not sufficient to allow the age specific ovarian cancer risks to be estimated in any more detail. Where the relative risks applied to two decades of age, we assumed that the ratio of incidence rates in those decades was the same as the ratio from the analysis of the Breast Cancer Linkage Consortium (Easton *et al*, 1995; Ford *et al*, 1998). The log-likelihood in this case was maximized over 16 parameters and the estimates are shown in Table 5

**Table 5:**
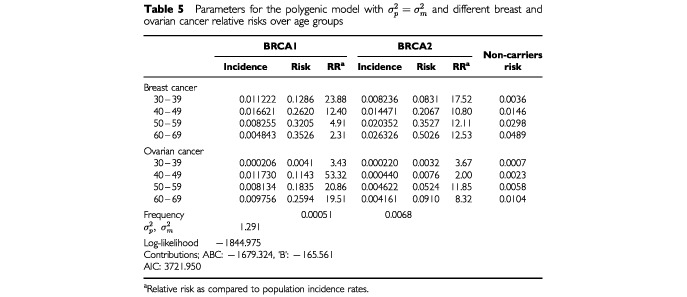
Parameters for the polygenic model with 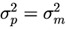 and different breast and ovarian cancer relative risks over age groups

together with the age specific incidence rates and cumulative risks. The model fits significantly better than the model with constant relative risk (*P*=0.03). The BRCA1 and BRCA2 mutation frequencies were estimated to be 0.051% (95% CI: 0.021–0.125%) and the BRCA2 mutation frequency was 0.068% (95% CI: 0.033–0.141%) respectively. The standard deviation for the polygenic and modifying effects was estimated to be 1.291 (95% CI: 1.097–1.519), identical to the model with constant relative risks. The estimated breast cancer incidence rates for BRCA1 increase up to the age group 40–49 years but then decrease, while the incidence rates for BRCA2 mutation carriers increase with age. The relative risk of breast cancer as compared with the population incidence rates decrease sharply with age for BRCA1 mutation carriers, from 23.88 in the 30–39 year age group to 2.31 in the age group 60–69 years (*P_trend_*=0.005). In contrast, the estimated breast cancer relative risk for BRCA2 mutation carriers as compared to population incidence was 17.52 for the age group 30–39 years but virtually constant over age 40 years at around 11–12 (*P_trend_*=0.697). The ovarian cancer incidence rates in BRCA1 carriers rise to a peak in the 40–49 years age group, followed by a slight decline, while the BRCA2 ovarian cancer incidence rates were highest in the 50–59 year age group.

The average risk of breast cancer for non-carriers, over all polygenic effects, is estimated to be 4.89% by age 70 years. Based on the 10 and 90% percentiles of the distribution of the polygenic component the cumulative breast cancer risks are 0.3 and 17.9% respectively. The cumulative breast cancer risk in BRCA1 mutation carriers over all modifiers is estimated to be 35.26% by age 70 years. This is lower than the estimate based on the Breast Cancer Linkage Consortium data of 71% (Easton *et al*, 1993). The estimated breast cancer risks at the 10 and 90% percentiles of the distribution of the modifying effect are 3.7 and 92.1% respectively. The average breast cancer cumulative risk by age 70 years in BRCA2 mutation carriers is 50.26% (7.2 and 99.3% at the 10 and 90% percentiles respectively). This is also lower than the BCLC estimate of 84% (Ford *et al*, 1998).

### Predicted number of mutations

The predicted number of mutations under the best fitting major gene and polygenic model (with a single relative risk parameter over all age groups) are shown in Table 2. The predicted numbers were very similar under the two models. The predicted number of mutations for the ABC families were similar to the observed number for index cases without family history of breast or ovarian cancer but they were slightly higher than observed for the index cases with positive family history of the diseases. For the ‘B’ families, the predicted number of BRCA2 mutations is very similar to the observed number, but the number of BRCA1 mutations is somewhat higher than predicted (21 *vs* 15.30 respectively for the polygenic model). The predicted number of mutations did not change much when separate relative risk parameters were assumed over all age groups for the polygenic model. In the ABC families, 10.44 BRCA1 mutations and 15.67 BRCA2 mutations were predicted (compared to eight and 15 observed respectively), while in the ‘B’ families 15.96 BRCA1 mutations and 16.72 BRCA2 mutations were predicted (compared to 21 and 18 observed respectively).

## DISCUSSION

We investigated the genetic models for familial breast cancer in a combined dataset consisting of both high-risk families and a population based series of breast cancer cases. We found the most parsimonious model to be one with a polygenic component with an equal variance in BRCA1/2 carriers and in non-carriers. This corresponds to a model in which several polymorphisms each act multiplicatively on risk (by for example, increasing the rate at which key mutational events will occur) to the same extent on carriers and non-carriers. A common recessive allele with moderate penetrance had the best fit among the major gene models fitted for ‘BRCA3’. However, the evidence for a major gene is weakened by the fact that the contribution of the ‘B’ families to the overall log-likelihood is always smaller under the major gene models for BRCA3 than their contribution under the sporadic model. On the other hand, the contribution of the ‘B’ families is larger under the polygenic models (with modifiers) than under the sporadic model.

Under the best fitting model (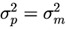), the frequency of BRCA1 mutations in the general population was estimated to be 0.051% (95% CI: 0.021–0.125%) which corresponds to about one in 974 individuals being a BRCA1 carrier. This is very similar to the estimate of Ford *et al* (1995) of one in 800. Higher estimates were obtained by Whittemore *et al* (1997) (0.14%) and Antoniou *et al* (2000) (0.13%). These studies were, however, based on segregation analysis of ovarian cancer and may be inflated by not allowing for other familial effects. The estimate from the ABC dataset alone was 0.023% (Antoniou *et al*, 2001), lower than the estimate from our combined analysis but included in the confidence interval. The mutation frequency for BRCA2 mutations was estimated to be 0.068% (95% CI: 0.033–0.141%). This corresponds to one in 734 individuals being a BRCA2 carrier in the general population. Again this is somewhat lower than 0.17% estimated in the ovarian cancer segregation analysis of Antoniou *et al* (2000) (0.17%). The frequency from the ABC analysis alone was again somewhat lower, 0.041% (Antoniou *et al*, 2001).

The fact that the observed number of BRCA1 mutations in the ‘B’ families was higher than predicted might suggest that we have slightly underestimated the BRCA1 allele frequency as a consequence of finding (by chance) too few BRCA1 mutations in the ABC study. Alternatively it may be that the sensitivity of mutation testing was lower in the much larger ABC study, although it is not clear why this should be true only for BRCA1.

Under our best fitting model the breast cancer risk for BRCA1 carriers, based on the average incidence over all modifying effects, was estimated to be 35.3% by age 70 years. This is moderately lower than the estimates derived by the BCLC studies of high risk families, which are in the range 71–85% (Easton *et al*, 1993, 1995) and also slightly lower than the estimates derived from risks to relatives of mutation carriers in population based series of breast cancer patients (Struewing *et al*, 1997; Hopper *et al*, 1999). These differences are broadly consistent with our best model, since cases in high risk families will be expected to have a much larger polygenic component than average, while relatives of early onset breast cancer cases will be expected to have only a slightly higher polygenic component than average. The ovarian cancer risk for BRCA1 mutation carriers is estimated to be 26% by age 70 years. This is somewhat higher than the estimate of Struewing *et al* (1997) but lower than the BCLC estimates (50.6–63.3%) (Easton *et al*, 1993, 1995). It is also lower than our previous estimate based on ovarian cancer families (66%) (Antoniou *et al*, 2000). The higher estimates from the last two studies are based on families that were selected on the basis of either ovarian cancer cases or number of breast and ovarian cancer cases, and could be the result of modifying genes or other familial factors increasing ovarian cancer risks. In principle, our model could be extended to account for such modifying factors.

The cumulative risk of breast cancer for BRCA2 mutation carriers was estimated to be 50.3% by age 70 years. Again this is lower than the BCLC estimates (Ford *et al*, 1998) but more similar to the estimates of the population based study of Hopper *et al* (1999). The corresponding ovarian cancer risk by age 70 years was estimated to be 9.1%. This is lower than the first BCLC estimate (27%) (Ford *et al*, 1998) and the estimate based on ovarian cancer families (31%) (Antoniou *et al*, 2000). It is also somewhat lower than the estimate from a recent BCLC study of genotype-phenotype correlations (14%) (Thompson and Easton, 2001) and the estimate of Struewing *et al* (1997) based on carriers of Ashkenazi Jewish founder mutations.

We found that the breast cancer incidence rates in BRCA1 mutation carriers relative to the population incidence rates decrease sharply with age. This contrasts with the pattern in BRCA2 carriers, in whom the relative risk is approximately constant over age 40 years. Thus, BRCA2 mutations are estimated to confer a breast cancer risk which is similar to the population risk but 10–12 times greater. This difference in the pattern of incidence rates is mirrored in the very different histopathology of tumours in BRCA1 and BRCA2 carriers (Lakhani *et al*, 1998), and reflects important mechanistic differences. A practical implication of these different age-incidence patterns, is that BRCA1 mutation carriers are more likely to develop breast cancer at a younger age than BRCA2 mutation carriers.

The polygenic model suggests that several genes, each having small but multiplicative effects on risk can account for the residual non-BRCA1/2 familial clustering of breast cancer. Genes other than BRCA1 and BRCA2 implicated in the aetiology of hereditary breast cancer include the TP53 gene (Malkin *et al*, 1990), and the PTEN gene (Nelen *et al*, 1996). Mutations in these genes, however, predispose to rare autosomal dominant syndromes and their contribution to familial aggregation is very small. Heterozygous carriers of the ataxia-talangiectasia gene ATM have been reported to be at increased risks of breast cancer (Swift *et al*, 1991; Easton, 1994; Olsen *et al*, 2001) and there is also evidence that rare HRAS1 alleles may be associated with moderate risks for breast cancer (Krontiris *et al*, 1993). It has been suggested that genes involved in steroid hormone metabolism and transport could act together defining a high-risk profile for breast cancer (Henderson and Feigelson, 2000). Genes included in this pathway include the HSD17B1 gene, the CYP17 and CYP19 genes and the Estrogen Receptor (ER) gene (Henderson and Feigelson, 2000). At present, however, there is no clear evidence that polymorphisms in these genes are associated with a significant risk (Dunning *et al*, 1998). In a meta-analysis of all the studies of low risk polymorphisms, significant evidence was found for carriers of the (TTTA)_10_ Cyp19 polymorphism, the GSTP1 Ile105Val polymorphism and the TP53 Arg72Pro polymorphism (Dunning *et al*, 1998) but the evidence was not conclusive.

Rebbeck *et al* (1999b) found that BRCA1 carriers with long repeat lengths of the (AG)_*n*_ polymorphism at the Androgen Receptor gene may have earlier age at diagnosis of breast cancer. Similarly, the steroid hormone metabolism gene AIB1 has been reported to modify the breast cancer risk in BRCA1 mutation carriers (Rebbeck *et al*, 1999a). Carriers of a variant AIB1 allele were found to have an age of diagnosis of breast cancer which is on average 3 years younger than non-carriers. However, neither of these results have been replicated.

The Hypergeometric Polygenic Model is equivalent to a fully additive polygenic trait, with no dominance or epistatic variance (Lange, 1997b). In practice some degree of dominance may exist, given the slightly higher familial relative risks among sibs (Pharoah *et al*, 1997). Among the major gene models fitted, the recessive model was the most parsimonious, and a recessive effect was also found by Cui *et al* (2001) in BRCA1/2 negative families. However, some of the evidence for a recessive effect may be due to secular increase in incidence, which would give rise to a higher risk to siblings of probands, and to the protective effect of parity which slightly reduces the risk to mothers of probands (Antoniou *et al*, 2001). It is also, of course, possible that some of the familial aggregation modelled here in terms of genetic susceptibility may be due to clustering of lifestyle risk factors (for example diet or reproductive factors) within families.

In conclusion, the present results suggest that a number of low penetrant genes may account for the familial clustering of breast cancer outside BRCA1 and BRCA2 families. The modifying effect on the breast cancer risks of BRCA1 and BRCA2 carriers may explain some of the differences between the risk estimates from population based studies and high risk families. The resulting model provides a framework for risk estimation to counsel women with a family history of breast cancer, allowing one to estimate carrier probabilities (separately for BRCA1 and BRCA2) and incidence rates in the same analysis. The model can also be extended straightforwardly to include risks of other cancers, second breast/ovarian cancers and tumour pathology. In principle it should also be possible to include lifestyle factors, although this would depend on assumptions about their effects in susceptible individuals.
